# Secretion of autoimmune antibodies in the human subcutaneous adipose tissue

**DOI:** 10.1371/journal.pone.0197472

**Published:** 2018-05-16

**Authors:** Daniela Frasca, Alain Diaz, Maria Romero, Seth Thaller, Bonnie B. Blomberg

**Affiliations:** 1 Department of Microbiology and Immunology, University of Miami Miller School of Medicine, Miami, FL, United States of America; 2 Department of Surgery, Division of Plastic and Reconstructive Surgery, University of Miami Miller School of Medicine, Miami, FL, United States of America; 3 Sylvester Comprehensive Cancer Center, University of Miami Miller School of Medicine, Miami, FL, United States of America; University of Palermo, ITALY

## Abstract

The adipose tissue (AT) contributes to systemic and B cell intrinsic inflammation, reduced B cell responses and secretion of autoimmune antibodies. In this study we show that adipocytes in the human obese subcutaneous AT (SAT) secrete several pro-inflammatory cytokines and chemokines, which contribute to the establishment and maintenance of local and systemic inflammation, and consequent suboptimal immune responses in obese individuals, as we have previously shown. We also show that pro-inflammatory chemokines recruit immune cells expressing the corresponding receptors to the SAT, where they also contribute to local and systemic inflammation, secreting additional pro-inflammatory mediators. Moreover, we show that the SAT generates autoimmune antibodies. During the development of obesity, reduced oxygen and consequent hypoxia and cell death lead to further release of pro-inflammatory cytokines, “self” protein antigens, cell-free DNA and lipids. All these stimulate class switch and the production of autoimmune IgG antibodies which have been described to be pathogenic. In addition to hypoxia, we have measured cell cytotoxicity and DNA damage mechanisms, which may also contribute to the release of “self” antigens in the SAT. All these processes are significantly elevated in the SAT as compared to the blood. We definitively found that fat-specific IgG antibodies are secreted by B cells in the SAT and that B cells express mRNA for the transcription factor T-bet and the membrane marker CD11c, both involved in the production of autoimmune IgG antibodies. Finally, the SAT also expresses RNA for cytokines known to promote Germinal Center formation, isotype class switch, and plasma cell differentiation. Our results show novel mechanisms for the generation of autoimmune antibody responses in the human SAT and allow the identification of new pathways to possibly manipulate in order to reduce systemic inflammation and autoantibody production in obese individuals.

## Introduction

The increase in the frequency of obesity is a worldwide phenomenon associated with several chronic diseases. These include cardiovascular disease (CVD) [1], Type-2 Diabetes (T2D) [2–4], cancer [5], psoriasis [6], atherosclerosis [7], and Inflammatory Bowel Disease [8]. The obesity pandemic affects all age groups and it has shown an increased prevalence over the past 20 years [9].

Obesity superimposed on aging appears to be an additional risk factor for older individuals, in which the prevalence of chronic diseases increases. We have previously shown that obesity decreases B cell responses in both young and elderly individuals [10]. To further elucidate our previously published work, we investigated if the adipose tissue was involved in the down-regulation of B cell function and antibody responses in young and elderly individuals and through which mechanism. It is known that aging induces a significant increase in adipose tissue (AT) mass and redistribution of body fat with increased Visceral Adipose Tissue (VAT) and ectopic VAT deposition [11, 12]. These are all strongly associated with poorer health conditions in elderly individuals, including the development of Insulin Resistance (IR) which also increases with age, as reviewed in [13].

Our prior studies in mice have shown that the VAT, which increases in size with aging, contributes to systemic and B cell intrinsic inflammation, reduced B cell responses and secretion of autoimmune antibodies. However, the specificity of these antibodies remains unknown [14]. The AT is not only a storage for excess nutrients but it is an active endocrine tissue [15]. Conversion of the AT from an insulin sensitive (IS) to an IR state during obesity involves expansion of adipocyte volume and remodeling of extracellular matrix components (collagens, elastins and the associated blood vasculature). This also involves a concomitant increase in the secretion of adipokines, pro-inflammatory cytokines and chemokines, which are involved in the recruitment of immune cells to the AT. Failure to undergo appropriate remodeling in response to over-nutrition is detrimental to body metabolic homeostasis, as excess nutrients promote meta-inflammation, or a low-grade systemic inflammation with the development of metabolic diseases. There is evidence that altered innate and adaptive immune responses occur in the “calorie-stressed” AT [15]. Immune cells are recruited to the murine AT by chemokines released by both adipocytes and infiltrating immune cells, generating a positive feedback loop, in which both the adipocytes and the infiltrating immune cells secrete pro-inflammatory mediators [14], contributing to both local and systemic inflammation via the circulating immune cells. These infiltrating immune cells become more inflammatory in the AT. We hypothesize that they would generate suboptimal immune responses in obese individuals by circulating to peripheral lymphoid organs.

In this study we have confirmed and extended to humans our results obtained in mice. We have identified several uninvestigated mechanisms through which the subcutaneous AT (SAT) induces the release of autoimmune IgG antibodies which have been described to be pathogenic [16]. We have evaluated several mechanisms (hypoxia, cell cytotoxicity, DNA damage) which may be responsible for the release of “self” antigens, which stimulate class switch and the production of autoimmune IgG antibodies. We have also measured the expression of transcription factors and membrane markers (T-bet and CD11c) responsible for the production of these antibodies in the SAT, as well as the release of cytokines known to promote isotype class switch.

## Materials and methods

### Study participants

Experiments were conducted using the obese SAT or blood (control) from the obese SAT of the same participants. SAT and blood were obtained from females undergoing breast reduction surgery (n = 12) or panniculectomy surgery (removal of lower abdominal fat) (n = 8) at the Division of Plastic and Reconstructive Surgery at the University of Miami Hospital. Study participants provided written informed consent. Study was reviewed and approved by the University of Miami Human Subject Research Office with Institutional Review Board IRB protocol #20160542, which reviews all human research conducted under the auspices of the University of Miami. Study participants were between 26 and 64 years of age, with Body Mass Index (BMI, kg/m^2^) 31–49. No significant differences were observed between the two groups of surgery patients and results obtained have been pooled together.

Participants without the following diseases or conditions known to alter the immune response were enrolled in the study: cancer, Congestive Heart Failure, Cardiovascular Disease, Chronic Renal Failure, renal or hepatic diseases, autoimmune diseases, infectious disease. Participants did not have recent (<3 months) trauma or surgery, pregnancy, or documented current substance abuse. Although our study has the limitation of including a limited number of participants, it has a unique advantage in recruiting individuals are without diseases/conditions which would alter the immune response. Most of the published reports, conversely, include diseases/conditions which may significantly impact the biological interpretation of the results.

### Isolation of the SAT

The SAT was harvested, weighed and washed with 1X Hanks’ Balanced salt Solution (HBSS). It was then resuspended in Dulbecco’s modified Eagle’s Medium (DMEM), minced into small pieces, passed through a 70 μm filter and digested with collagenase type I (SIGMA C-9263) for 1 hr in a 37°C water bath. Digested cells were passed through a 300 μm filter, centrifuged at 300 g in order to separate the floating adipocytes from the stromal vascular fraction (SVF) containing the immune cells. The cells floating on the top were transferred to a new tube as adipocytes. The cell pellet (SVF) on the bottom was resuspended in ACK for 3 min at RT (room temperature) to lyse the Red Blood Cells. Both adipocytes and SVF were washed 3 times with DMEM. Adipocytes were sonicated for cell disruption in the presence of TRIzol, and then centrifuged at 1000 x g at 4°C for 20 min to separate the soluble fraction from the lipid and cell debris. Soluble fraction was then used for RNA isolation. Adipocytes and SVF were also lyzed to obtain protein extracts or they were used for cell culture.

### Preparation of adipocyte-conditioned medium (ACM)

A piece of freshly harvested SAT was also cultured in DMEM for 24 hrs at the concentration of 1 g/100 μl to obtain ACM as previously described [17]. ACM was used to stimulate immune cells. Pro-inflammatory cytokine content in ACM was measured by the Cytometric Bead Array (CBA) (BD 560484), according to the manufacturer’s instructions. Samples were acquired on an LSRII (BD) and analyzed using FlowJo 10.0.6 software.

### Culture of naïve B cells in the presence of ACM

Naïve B cells were sorted from the peripheral blood of 8 lean healthy individuals and stimulated in the presence of ACM for 5 and 8 days to detect mRNA expression of activation-induced cytidine deaminase (AID) and BLIMP-1, respectively. B cells (10^6^/ml of ACM) were stimulated with 5 μg/ml of CpG. Results show qPCR values (2^-ΔΔCt^) of RNA expression of these transcription factors. DMEM was used as negative control.

### Flow cytometry

One hundred μl of blood from the obese SAT or 5x10^5^ cells from the SVF were stained for 20 min at room temperature with the following antibodies: Live/Dead detection kit (InVitrogen1878898), anti-CD45 (Biolegend 368540), anti-CD19 (BD 555415), anti-CD3 (BD 555339), anti-CD4 (BD 550631), anti-CD8 (BD 560179), anti-TCRαβ (Biolegend 306717), anti-TCRγδ (Biolegend 331221), to evaluate frequencies of the major lymphocyte populations; anti-TCRγδ1 (Thermo Fisher Scientific TCR2730) and anti-TCRγδ2 (Biolegend 331407) to evaluate the frequencies of TCRγδ cell subsets; anti-CD16 (Biolegend 302017) and anti-CD56 (BD 555516) to evaluate the frequencies of NK cells; anti-CD14 (BD 555399) to evaluate the frequencies of monocytes and macrophages (MΦ). To evaluate B cell subsets, blood and SVF were stained with anti-CD19, anti-CD27 (BD 555441) and anti-IgD (BD 555778) to measure naive (IgD+CD27-), IgM memory (IgD+CD27+), switched memory (swIg, IgD-CD27+), and late memory (LM, IgD-CD27-) B cells.

After staining, red blood cells were lyzed using the RBC Lysing Solution (BD 555899), according to the manufacturer’s instructions. Up to 10^5^ events in the lymphocyte gate were acquired on an LSR-Fortessa (BD) and analyzed using FlowJo 10.0.6 software. Single color controls were included in every experiment for compensation. Isotype controls were also used in every experiment to set up the gates.

To identify Germinal Center (GC) B cells, blood and SVF cells were stained with Live/Dead detection kit, anti-CD45, anti-CD19, anti-IgD, and anti-CD10 (Biolegend 312225). GC B cells were CD10+IgD-. GC B cells were also stained for the detection of intracellular Bcl-6 (Biolegend 358511). To identify GC T cells, SVF cells were stained with anti-CD3, anti-CD4, anti-PD1 (Biolegend 329907) and anti-CXCR5 (Biolegend 356925). GC T cells were CD3+CD4+PD1+CXCR5+.

### NK cytotoxicity

NK cytotoxicity was measured by flow cytometry, using a protocol previously described [18–20]. Briefly, PBMC and SVF cells in a 96-well plate, at the concentration of 1.5×10^6^ cells/ml (200 μl final volume), were stained with anti-CD16 (Biolegend 302017) for 15 min at rt. Then, anti-CD107a (Biolegend 328619) was added. For each individual a positive control containing PMA and ionomycin at a final concentration of 50 ng/ml and 1 μg/ml (respectively) was included, as well as a negative control without stimuli, to measure spontaneous stimulation. For stimulation with the K562 cell line (positive control), or with SAT MΦ, we used the target:NK cell ratio of 1:1. This ratio was determined by estimating NK cell frequencies in blood and SVF. The plate was incubated at 37°C for 1hr. After this time, Golgistop (0.67 μl/ml) and brefeldin A (1 μg/ml) were added for an additional 4 hrs. Following incubation, cells were stained with anti-CD56 and anti-CD3, fixed, permeabilized, stained intracellularly with anti-IFN-γ (Biolegend 502539) for 30 min and run on an LSR-Fortessa (BD) and analyzed using FlowJo 10.0.6 software. Single color controls were included in every experiment for compensation. Isotype controls were also used in every experiment to set up the gates.

### PrimeFlow RNA assay

PrimeFlow RNA assay allows the simultaneous measurement of intracellular mRNA and proteins in specific cells at the single cell level by an amplified Fluorescence In Situ Hybridization technique, in combination with flow cytometry. Briefly, PBMC or SVF were left unstimulated or they were stimulated with the TLR7 agonist CL097 (single stranded RNA) at the concentration of 5 μg/10^6^ cells. At the end of the stimulation time, cells were labeled with Live/Dead detection kit, anti-CD45 and anti-CD19. For T-bet mRNA detection, target probe hybridization was performed using type 1 (AlexaFluor647) probe for T-bet (Affymetrix VA1-16417-06). Negative control was the sample without the target probe. Cells were incubated for 2 hrs with the probe in a precisely calibrated incubator set to 40°C. All samples were then incubated with the PreAmplification (PreAmp) reagent for 1.5 hrs and the Amplification (Amp) reagent for an additional 1.5 hrs at 40°C. After signal amplification, cells were incubated with the label probe at 40°C for 1 hr. Cells were washed and suspended in staining buffer prior to acquisition. Approximately 10^5^ events were acquired from each sample on an LSR-Fortessa (BD) and analyzed using FlowJo 10.0.6 software. Spectral compensation was completed using single color control samples. Isotype controls were also used in every experiment to set up the gates.

To measure immune activation markers on T-bet expressing B cells in PBMC and SVF, cells were additionally membrane stained with anti-CD11c (Biolegend 301625).

### RNA extraction and quantitative (q)PCR

After sorting, cells were resuspended in TRIzol (Ambion) (10^6^ cells/100 μl), then RNA extracted for quantitative (q)PCR. Total RNA was isolated according to the manufacturer’s protocol, eluted into 10 μl distilled water and stored at -80°C until use. Reactions were conducted in MicroAmp 96-well plates and run in the ABI 7300 machine. Calculations were made with ABI software. Briefly, we determined the cycle number at which transcripts reached a significant threshold (Ct) for each target gene and for GAPDH as control. A value of the target gene, relative to GAPDH, was calculated and expressed as ΔCt. Reagents and primers (Taqman) were from Life Technologies.

### Preparation of protein lysates

Adipocytes and SVF were centrifuged in a 5415C Eppendorf microfuge (2,000 rpm, 5 min). Total cell lysates were obtained using the M-PER (Mammalian Protein Extraction Reagent, Thermo Scientific), according to the manufacturer’s instructions. Aliquots of the protein extracts were stored at -80°C. Protein content was determined by Bradford assay [21].

### Western blotting (WB)

Protein extracts at equal protein concentration were denatured. Then they were electro-transferred onto nitrocellulose filters. Filters were incubated with the following primary antibodies: anti-phospho-Hormone Sensitive Lipase (HSL) (ThermoFisher PA5-17488, 1:1000 diluted), anti-phospho-H2AX (Cell Signaling 9718S, 1:500 diluted), in PBS-Tween 20 containing 5% milk. The primary antibodies anti-HSL (ThermoFisher PA5-26383, 1:1000 diluted) and anti-H2AX (Cell Signaling 2595, 1:1000 diluted) were used as loading controls. After overnight incubation with the primary antibodies, immunoblots were incubated with HRP-conjugated secondary antibodies (from Jackson ImmunoResearch Laboratories) for 1.5 hrs at 4°C. Membranes were developed by enzyme chemiluminescence and exposed to CL-XPosure Film (Pierce). Films were scanned and analyzed using the AlphaImager Enhanced Resolution Gel Documentation & Analysis System (Alpha Innotech). Images were quantitated using the AlphaEaseFC 32-bit software. Phospho proteins were detected first. Membranes were stripped and reblotted to detect unphosphorylated proteins.

### Mitochondrial respiration

Mitochondrial respiration was measured using a protocol originally described [22]. Briefly, PBMC or SVF cells (4x10^6^ in 1.5 ml) were washed in an Eppendorf tube with permeabilized-cell respiration buffer (PRB) containing 0.3M mannitol, 10mM KCl, 5mM MgCl_2_, 0.5mM EDTA, 0.5mM EGTA, 1 mg/ml BSA and 10mM KH_3_PO_4_ (pH 7.4). Then cells were air-equilibrated at 37°C in PRB supplemented with 10 U hexokinase (in order to promote synthesis of glucose-6-phosphate and thus prevent accumulation of ATP), and 2mM ADP. Cells were immediately loaded into a polarographic chamber with a Hamilton syringe and endogenous respiration was measured as follows. Freshly prepared digitonin (60 μg/10^6^ cells) was added to permeabilize cells. Then respiration measurements in permeabilized cells were performed by adding 5mM glutamate + 5mM malate, then 5mM succinate. Respiration was inhibited by adding 3 μl of 80mM KCN.

### Autoimmune antibody secretion in the SAT

Immune cells in the SVF were counted and cultured for 10–12 days in complete RPMI (RPMI 1640, supplemented with 10% FCS, 10 μg/ml Pen-Strep, 1mM Sodium Pyruvate, and 2x10^-5^ M 2-ME and 2mM L-glutamine) at the concentration of 2x10^6^ cells/ml. Cells were either left unstimulated or they were stimulated with CpG (10 μg/ml). Supernatants were collected and stored at -80°C.

### ELISA to detect IgG in culture supernatants

Total IgG in SVF culture supernatants were measured by Bethyl kit E80-104, according to the manufacturer’s instructions.

### Specificity of the autoimmune antibodies secreted in the SAT

To measure fat-specific IgG in SVF culture supernatants, protein lysates from adipocytes were prepared as indicated above and used for coating the ELISA plates (at the concentration of 10 μg/ml). Detection antibody was an HRP-labelled goat anti-human IgG-Fc antibody (Bethyl A80-104P-71). Supernatants were enriched in IgG using Protein G Dynabeads (ThermoFisher Scientific 10007D).

### Statistical analyses

Mean comparisons were performed by paired/unpaired Student’s t test test (two-tailed), using GraphPad Prism version 5 software, which was used to construct all graphs.

## Results and discussion

### The obese SAT is heavily infiltrated with pro-inflammatory immune cells

Data fom obese mice and humans have clearly indicated that the hypertrophied AT becomes heavily infiltrated by a variety of immune cells. Monocyte infiltration and MΦ expansion within the AT has been considered a major driver of inflammation, due to the secretion of pro-inflammatory cytokines and chemokines involved in the recruitment of immune cells to the AT [23]. However, our data below show that other immune cell types infiltrate the human obese SAT and contribute to the establishment of inflammatory conditions responsible for IR.

**Fig 1:**
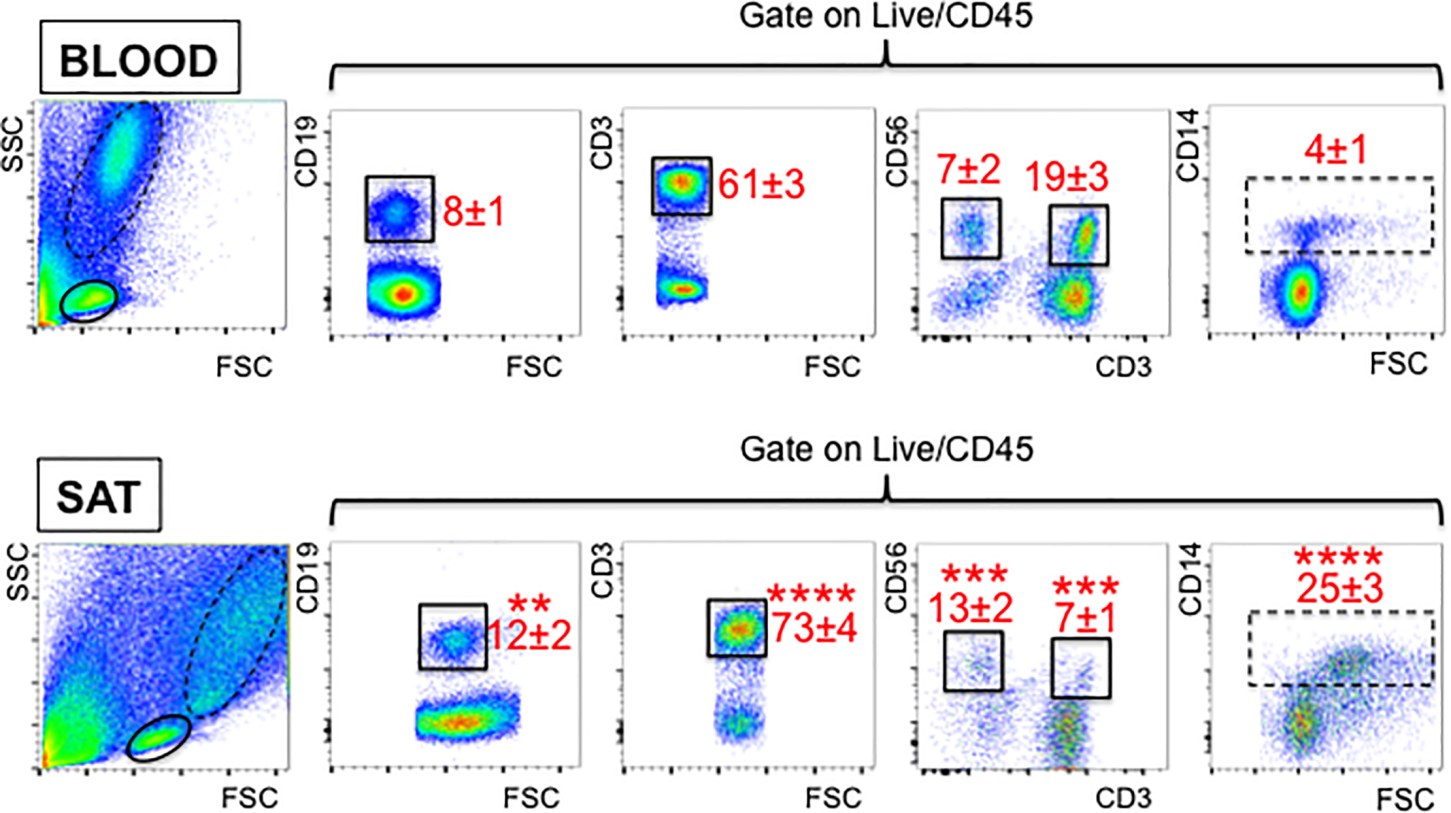
Frequencies of immune cells in the obese SAT *versus* blood. The blood and the SVF from the SAT of the same individuals (n = 20, representing all individuals recruited) were stained to evaluate the frequencies of B, T, NK, NKT as well as monocytes and MΦ. Gating strategies and a representative dot plot from one individual are shown. B, T, NK, NKT are gated on the continuous line, whereas monocytes and MΦ are gated on the dotted line. Means±SE are shown in red. Mean comparisons between groups were performed by Student’s t test (two-tailed). **p<0.01, ***p<0.001, ****p<0.0001.

We analyzed immune cell infiltration into the SAT of recruited participants by flow cytometry. Fig 1 shows our data comparing SAT *versus* blood from the same individuals. Briefly, we have measured B cells (CD19+), T cells (CD3+), NK (CD56+), NKT (CD56+CD3+), monocytes and MΦ (CD14).

**Fig 2:**
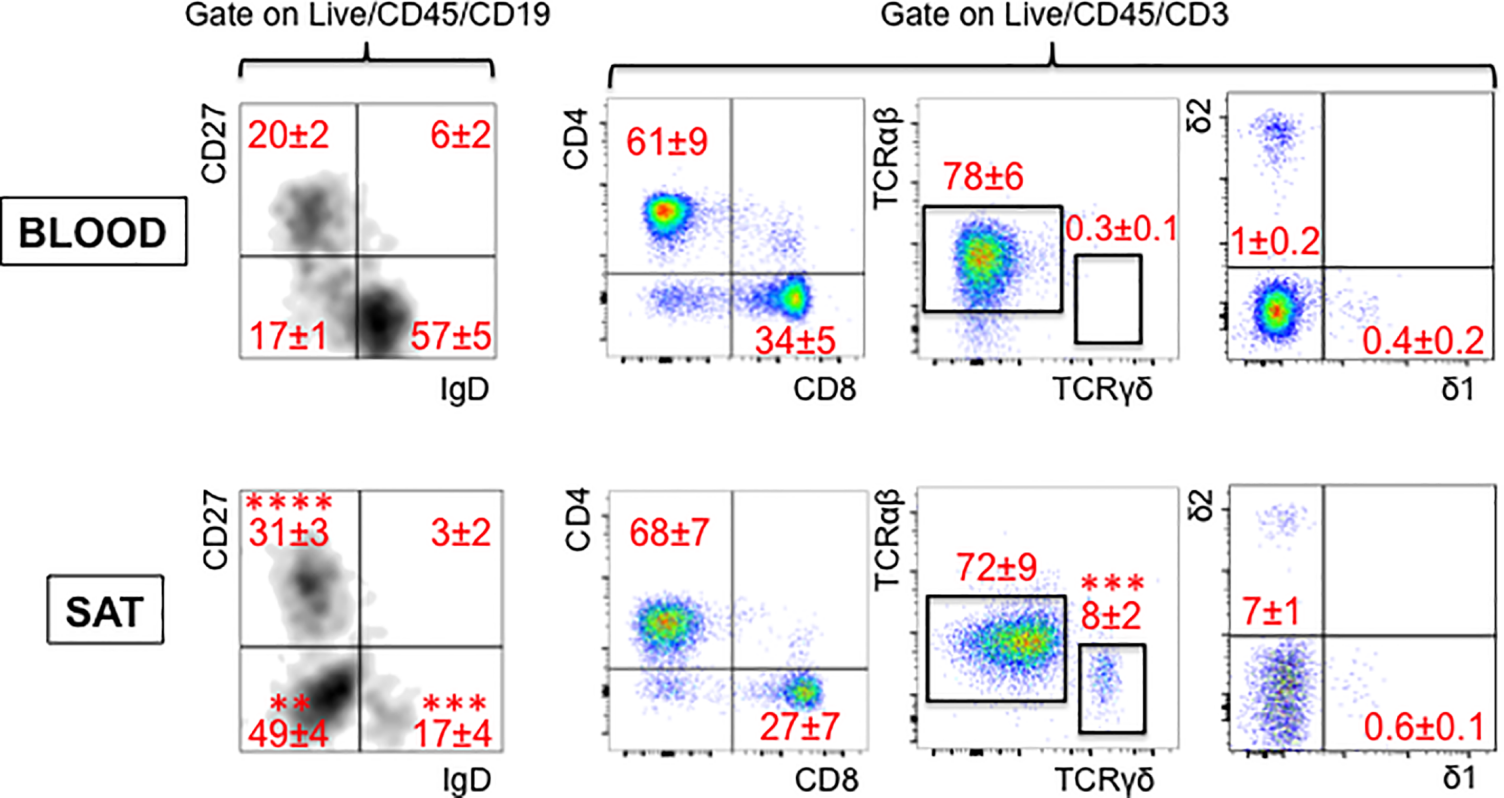
Frequencies of B and T cell subsets in the obese SAT *versus* blood. The blood and the SVF from the SAT of the same individuals were stained to measure B (left) and T (right) cell subsets. Gating strategies and a representative dot plot from one individual are shown. Means±SE are shown in red. Mean comparisons between groups were performed by Student’s t test (two-tailed). **p<0.01, ***p<0.001, ****p<0.0001.

Fig 2 (left) shows the major B cell subsets: naive (IgD+CD27-), IgM memory (IgD+CD27+), switched memory (IgD-CD27+) and late memory (LM, IgD-CD27-), also known as double negative, tissue-like or atypical memory B cells. Fig 2 (right) shows T cell subsets (CD4/CD8, TCRαβ/TCRγδ, γδ1/γδ2).

Results in Figs 1 and 2 show increased percentages of pro-inflammatory immune cells in SAT as compared to the blood from the same individuals, i.e. memory B cells (in particular switched and LM B cells), TCRγδ T cells, NK and monocytes and MΦ. Conversely, NKT cell percentages were found significantly decreased. We also measured frequencies of γδ1 *versus* γδ2 subsets in blood and in the SAT. Results indicate that the majority of TCRγδ T cells in SAT and blood are γδ2, but the ratio γδ2:γδ1 is higher in the SAT, as expected as γδ2 T cells are more pro-inflammatory [24, 25].

### The infiltration of immune cells is due to secretion of pro-inflammatory chemokines in the SAT

**Fig 3:**
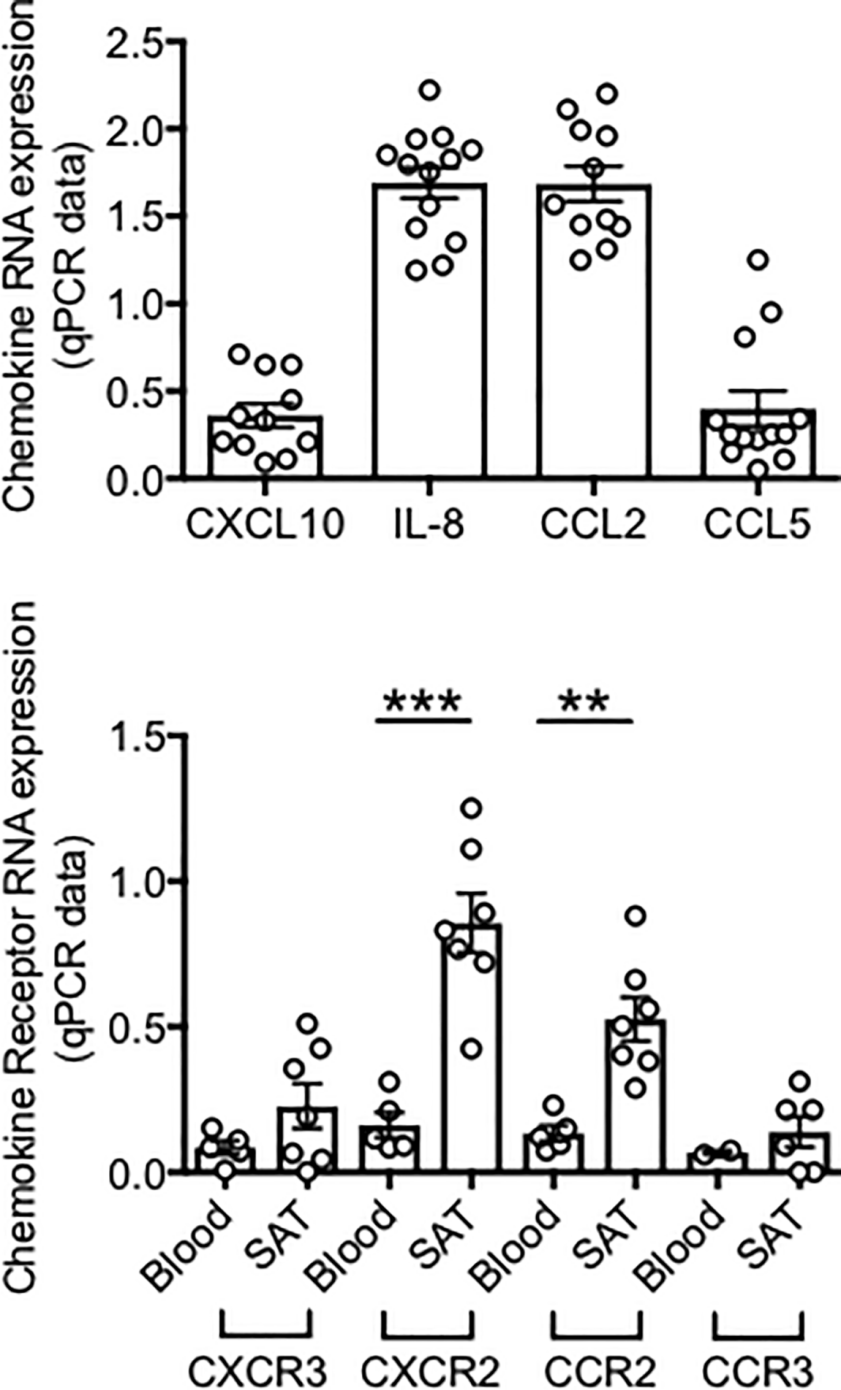
RNA expression of chemokines in adipocytes and corresponding chemokine receptors in the obese SAT *versus* blood. **Top.** Adipocytes (AD) were sonicated for cell disruption in the presence of TRIzol to separate the soluble fraction (used for RNA isolation) from lipids and cell debris. Results show qPCR values (2^-ΔΔCt^) of CXCL10, IL-8, CCL2, CCL5 RNA expression. **Bottom.** The SVF were resuspended in TRIzol. AD and SVF were from the same obese individuals. PBMC (blood) were from obese individuals age-, gender- and BMI-matched. Results show qPCR values (2^-ΔΔCt^) of CXCR2, CXCR3, CCR2, CCR3 RNA expression. Mean comparisons between groups were performed by Student’s t test (two-tailed). **p<0.01, ***p<0.001.

We hypothesize that the adipocytes (as well as immune cells in the SVF) secrete pro-inflammatory chemokines which promote migration of immune cells to the SAT. We tested RNA expression of chemokines in adipocytes and we selected chemokines known to attract different types of immune cells, including B cells, to the AT. Results in Fig 3 (top) show that SAT adipocytes express RNA for the chemokines CXCL10, IL-8, CCL2, CCL5. We found the corresponding receptors (CXCR3, CXCR2, CCR2, CCR3) expressed by the immune cells in the SVF. Highest levels of expression were found for CXCR2, the receptor for IL-8 (Fig 3, bottom). We also measured RNA expression of these receptors in PBMC (blood) from obese individuals age-, gender- and BMI-matched. Results show higher expression in SVF *versus* PBMC, suggesting that immune cells may infiltrate the obese SAT. As for B cells, the chemokine receptors CXCR3, CXCR2, CCR2 are expressed on both naïve and memory B cells, whereas CCR3 is only expressed on memory B cells [26], suggesting that all B cell subsets may infiltrate the obese SAT.

### The adipocytes also secrete many pro-inflammatory cytokines and in higher amounts as compared to the immune cells in the SVF and in blood

Adipocytes in the SAT are highly inflammatory and secrete several pro-inflammatory cytokines and chemokines, in addition to adipokines (leptin, adiponectin) involved in the regulation of metabolic homeostasis [15, 23]. All secreted cytokines, chemokines, adipokines contribute to the establishment and maintenance of local and systemic inflammation.

**Fig 4:**
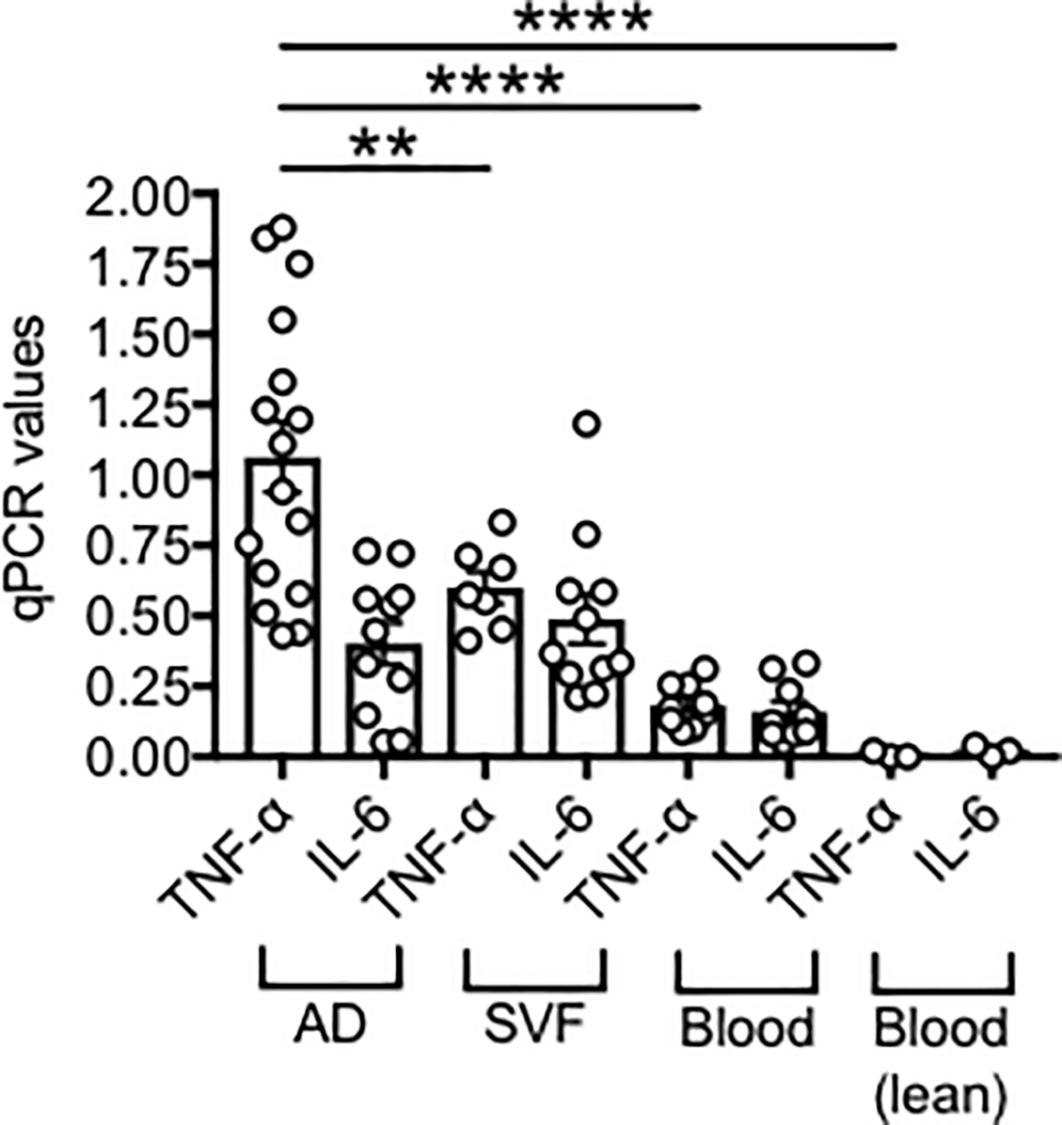
RNA expression of pro-inflammatory cytokines in the obese SAT *versus* blood. Adipocytes (AD), SVF and PBMC (blood) were sonicated for cell disruption in the presence of TRIzol to separate the soluble fraction (used for RNA isolation) from lipids and cell debris. AD and SVF were from the same obese individuals. PBMC (blood) were from obese individuals age-, gender- and BMI-matched. Results show qPCR values (2^-ΔΔCt^) of TNF-α and IL-6 RNA expression.

Fig 4 shows our data on TNF-α and IL-6 RNA expression in adipocytes from SAT samples, as compared to SVF from the same individuals, and to PBMC (blood) from obese individuals age-, gender- and BMI-matched. We also show TNF-α and IL-6 RNA expression in PBMC from lean individuas age- and gender-matched as negative controls. Results demonstrate significantly higher expression of TNF-α RNA in adipocytes *versus* SVF, whereas expression of IL-6 RNA is comparable in adipocytes and SVF. These findings suggest that both the adipocytes and the infiltrating immune cells would need to be targeted to reduce the levels of local inflammation and decrease the pathogenic function of B cells. Levels of these cytokines are lower in the blood from obese individuals and even more in the blood from lean individuals in which no expression of TNF-α and/or IL-6 was detected.

### TNF-α released in the SAT is a regulator of inflammation, lipolysis and oxidative stress

The mechanisms responsible for high levels of TNF-α in the SAT are not completely clear. Hyperinsulinemia, which is usually associated with obesity, could be a relevant factor [27]. TNF-α secreted in the obese AT, more than systemic TNF-α, is associated with IR [28]. Moreover,TNF-α released in the obese AT is a tonic regulator of lipolysis, the process of hydrolysis of tryglycerides to generate free fatty acids (FFAs) and lipids [29]. It has been proposed that adipocyte-derived TNF-α contributes to elevated levels of FFAs in the blood of obese individuals [29], and neutralization of TNF-α *in vivo* in obese mice decreases circulating levels of FFAs [30]. Both FFAs and lipids induce inflammation through NF-kB activation [31], activation of pro-inflammatory MΦ and IR [32, 33]. FFAs also directly inhibit B cell function (cytokine production) *in vitro* [34].

We have not performed any lipidomic analysis in our study. We believe that these analyses are relevant to identify specific lipid signatures of the subcutaneous AT to identify differences between lean and obese tissues or, alternatively, differences between subcutaneous and visceral AT. We also believe that specific lipid signatures may represent powerful approaches for clinical diagnosis, and could be done in future studies, but these were not the purpose of this current study.

**Fig 5:**
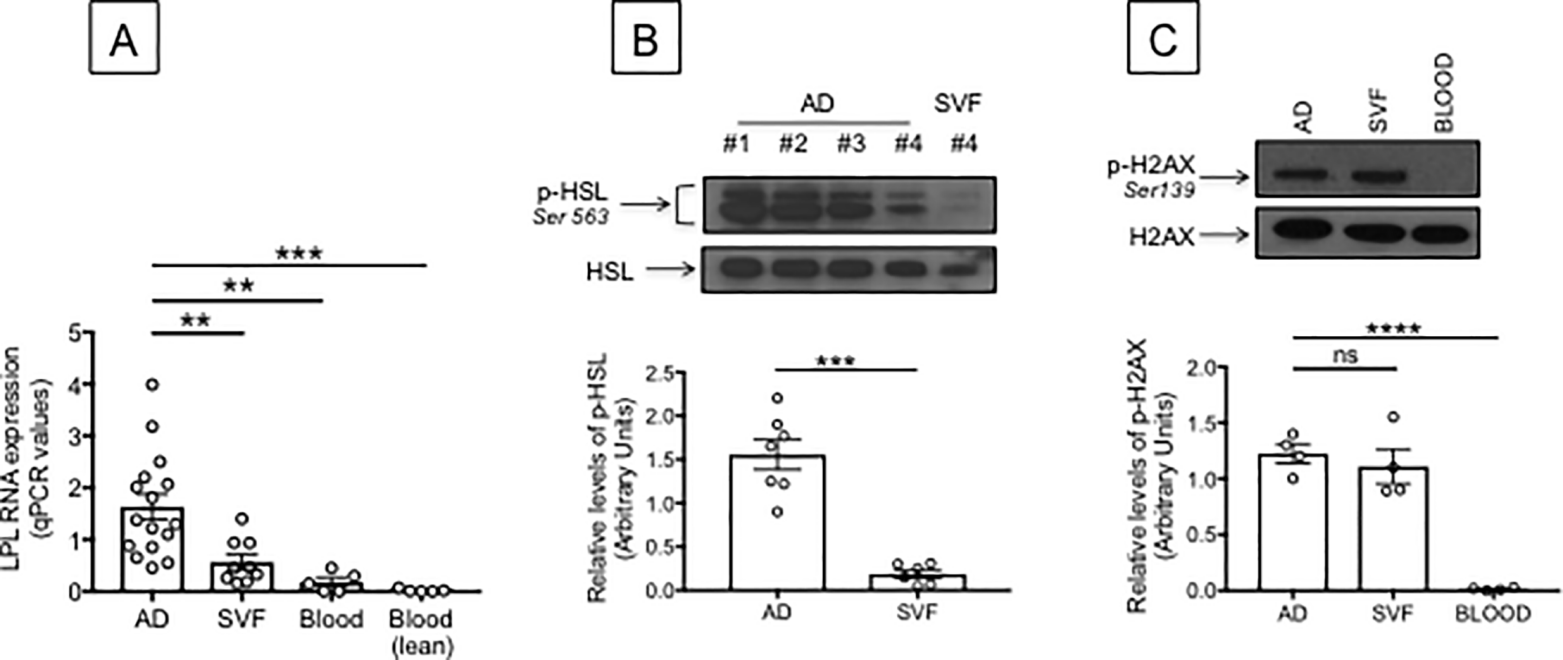
Measure of lipolysis in the obese SAT *versus* blood. **A.** Adipocytes (AD), SVF and PBMC (blood) were sonicated for cell disruption in the presence of TRIzol to separate the soluble fraction (used for RNA isolation) from lipids and cell debris. AD and SVF were from the same obese individuals. PBMC (blood) were from obese individuals age-, gender- and BMI-matched. Results show qPCR values (2^-ΔΔCt^) of LPL RNA expression. **B.** Total protein lysates of adipocytes (AD) and SVF (from different obese individuals age-, gender- and BMI-matched) were prepared and run in WB to measure phospho-HSL. A representative WB for the higest and lowest values is shown (top). Mean comparisons between groups were performed by Student’s t test (two-tailed). ***p<0.001. **C.** Total protein lysates of adipocytes (AD) and SVF (same as in B) were prepared and run in WB to measure phospho-H2AX. Total protein lysates of PBMC (blood) from different obese individuals age-, gender- and BMI-matched, were also prepared and run in WB. A representative WB is shown (top). Mean comparisons between groups were performed by Student’s t test (two-tailed). ****p<0.0001.

We have measured markers of adipocyte lipolysis such as lipoprotein lipase (LPL) and phospho-Hormone-Sensitive Lipase (HSL). LPL, present in the capillary bed, hydrolyzes tryglycerides or lipids present in circulating lipoproteins [35]. HSL, present on the surface of cells that line capillaries in the AT, hydrolyzes triacylglycerols, diacylglycerols, monoacylglycerols and cholesteryl esters, as well as other lipid and water soluble substrates. Its activity is regulated post-translationally by phosphorylation [36]. Fig 5A shows higher levels of LPL RNA expression in adipocytes, as compared to SVF from the same individuals, and to blood from obese individuals age-, gender- and BMI-matched. We also show LPL RNA expression in PBMC from lean individuas age- and gender-matched as negative controls. The higher lipolysis in adipocytes *versus* SVF is confirmed in Fig 5B showing that phospho-HSL levels are also higher in adipocytes *versus* SVF. These results altogether suggest that TNF-α released in the obese SAT may be a chronic and tonic regulator of lipolysis, leading to continuous release of FFAs and glycerol, as previously shown and reviewed in [29].

Excessive lipolysis in the SAT leads to increased oxidative stress that promotes DNA damage and SAT inflammation. We measured the phosphorylation of the histone H2AX as a measure of DNA damage in the SAT and in PBMC (blood) as control. Phosphorylation of H2AX plays a key role in the cellular DNA damage response and is required for the assembly of DNA repair proteins at the sites containing damaged chromatin as well as for activation of checkpoint proteins involved in cell cycle arrest [37]. Results in Fig 5C show similar levels of phospho-H2AX in adipocytes and SVF cells, suggesting that DNA damage is occurring in the SAT, as opposed to the blood where no detectable DNA damage was observed in total lysates of unstimulated PBMC.

### Mechanisms for the release of “self” antigens in the SAT

**Fig 6:**
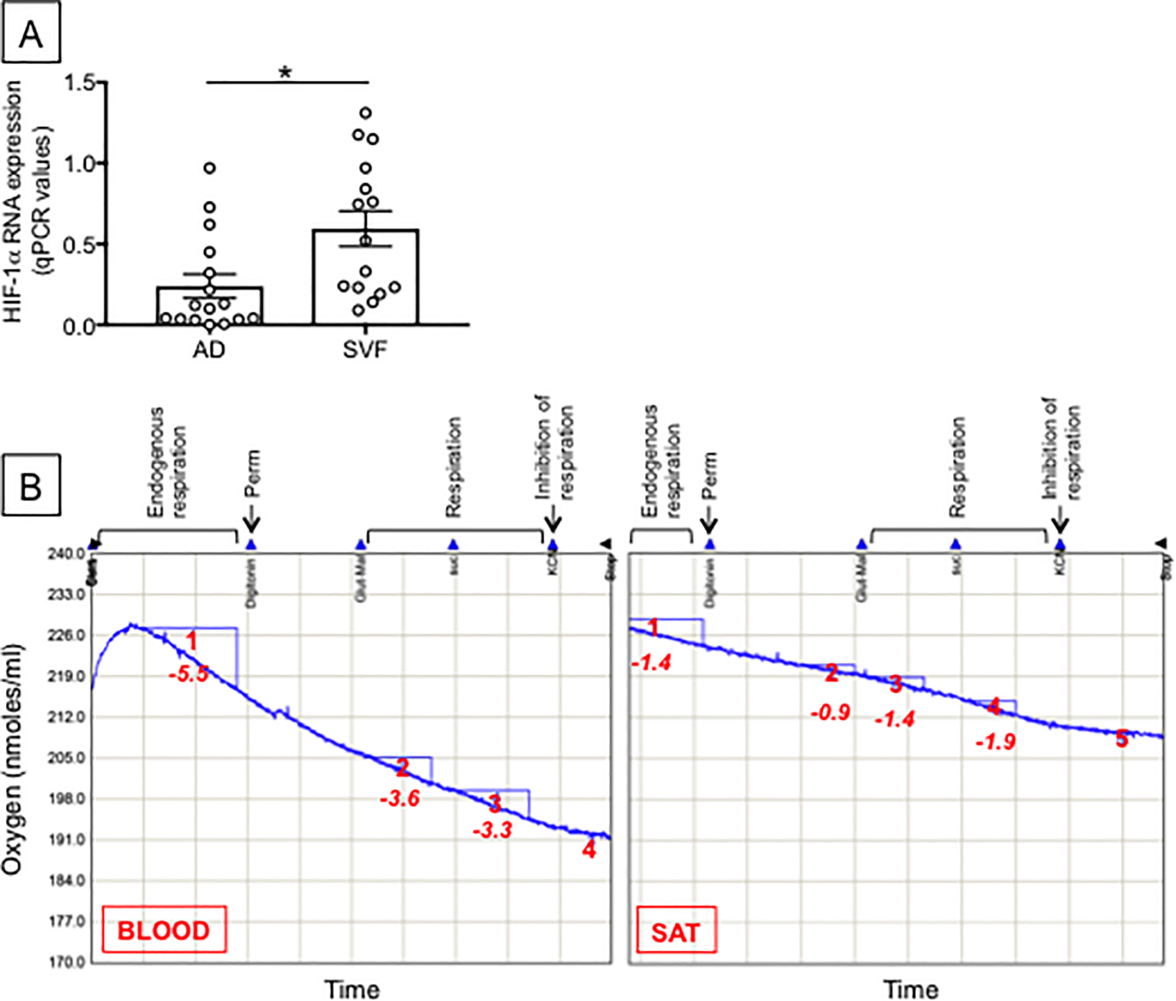
Measure of hypoxia in the obese SAT *versus* blood. **A.** Hypoxia was measured by RNA expression of HIF-1α in adipocytes (AD) and SVF from the same individuals. AD were sonicated for cell disruption in the presence of TRIzol. The SVF was also resuspended in TRIzol. AD and SVF were from the same individuals. Results show qPCR values (2^-ΔΔCt^) of HIF-1α RNA expression. Mean comparisons between groups were performed by Student’s t test (two-tailed). *p<0.05. **B.** Respiration in the SVF and in PBMC (blood) from different obese individuals age-, gender- and BMI-matched was measured as described in Materials and methods. Numbers in red indicate Oxygen consumption rates (O_2_ nmoles/ml/min) in the example shown. Results are representative of 2 independent experiments.

Many mechanisms may account for the release of “self” antigens in the SAT. We measured hypoxia as one of these mechanisms because, during the development of obesity, the supply of oxygen to the expanding AT becomes inadequate. As a consequence, areas of hypoxia and cell death are generated [38, 39] with further release of pro-inflammatory cytokines [40]. We hypothesize that hypoxia-driven cell death in the obese SAT induces the release of “self” antigens. Moreover, studies in mice have clearly indicated that the hypoxic SVF is enriched in IgG2c antiibodies [41], which have been shown to be pathogenic and autoimmune [16]. We evaluated the expression of the transcription factor hypoxia-inducible factor-1α (HIF-1α) in adipocytes and SVF. This transcription factor is activated by hypoxia. Results in Fig 6A show that RNA expression of HIF-1α is detectable in both adipocytes and SVF, but it is higher in the SVF, indicating that the SVF is the major site for the hypoxic response.

Because we found higher levels of HIF-1α RNA in the SVF, we then measured mitochondrial respiration in the SVF. Mitochondria consume the largest amount of oxygen in cells to allow oxidative phosphorylation, which represents the primary metabolic pathway to generate ATP. In hypoxic conditions, ATP generation is reduced which could result in cell death [42]. Results (Fig 6B) show that the rate of mitochondrial respiration in the SVF was significantly reduced as compared to that in PBMC (blood), suggesting reduced efficiency of ATP production in immune cells in the SAT *versus* those in blood.

**Fig 7:**
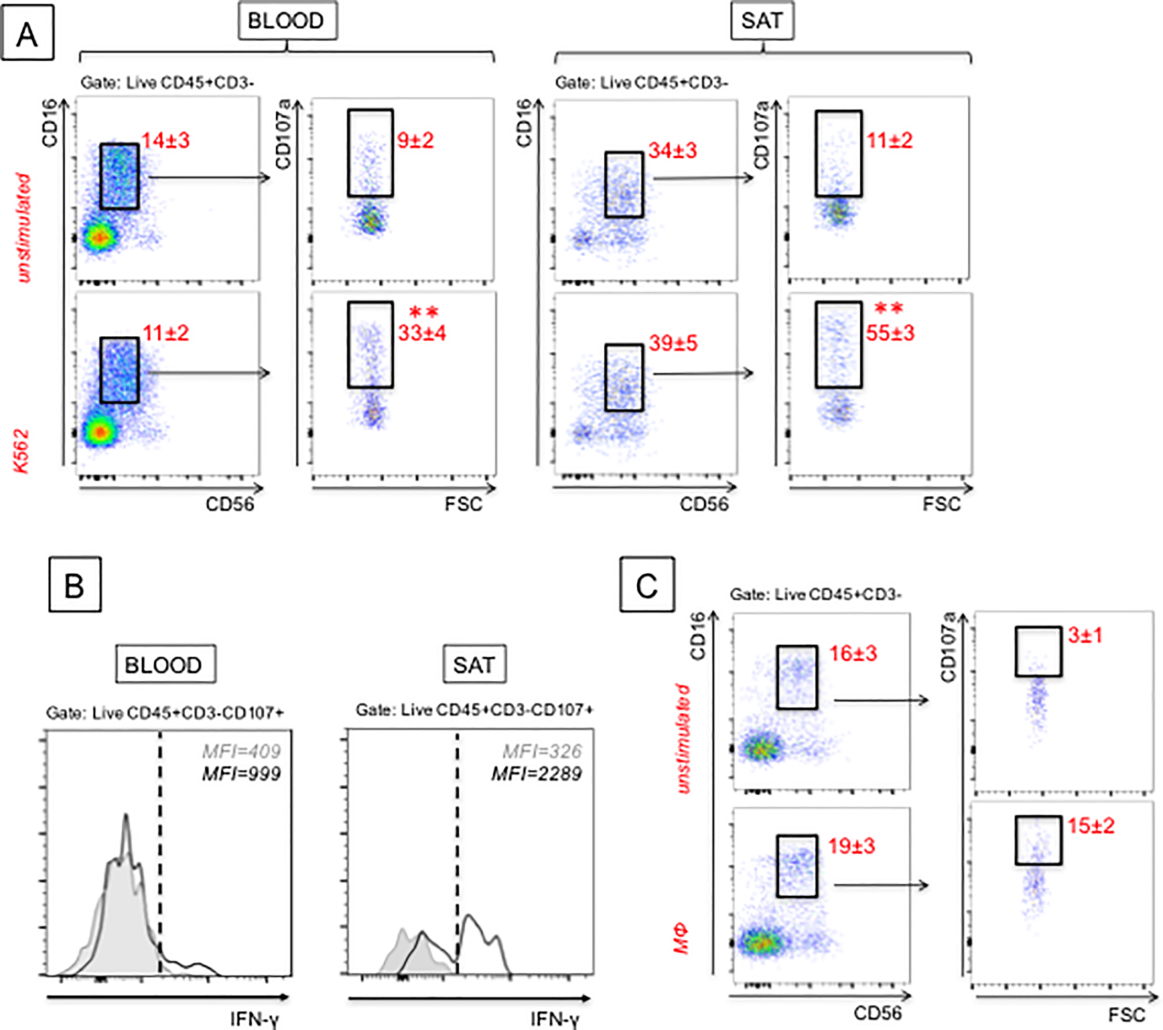
NK degranulation is higher in SAT as compared to blood. **A.** Cytotoxicity of CD16+CD56^dim^ NK cells from the SVF and PBMC (blood) as control was measured against the leukemic cell target K562. PBMC are from different obese individuals age-, gender- and BMI-matched. Numbers in red are means±SE from 3 independent experiments. The target:NK cell ratio was 1:1. Mean comparisons between groups were performed by Student’s t test (two-tailed). **p<0.01. **B.** IFN-γ was detected by intracellular staining and flow cytometry, before (grey line, filled histogram) and after (black line, unfilled histogram) stimulation with K562, overnight at 37°C. **C.** Cytotoxity of CD16+CD56^dim^ NK cells was measured against SAT MΦ. SAT MΦ were enriched from the SVF of the same individuals through plastic adherence for 1 hr at 37°C. Numbers in red are means±SE from 3 independent experiments. The target:NK cell ratio was 1:1.

Next, we measured NK cytotoxicity as an additional mechanism to induce cell death in the SAT and release of “self” antigens. We measured NK cytotoxicity, rather than CD8 T cell cytotoxicity, because NK cells were found significantly increased in frequencies in the SAT as compared to the blood, whereas frequencies of CD8 T cells were comparable in SAT and blood. NK cytotoxicity was measured by flow cytometry. Briefly, the test is based on the expression of CD107a by human NK cells, a marker of degranulation, significantly up-regulated on the membrane of NK cells following stimulation with PMA/Ionomycin. This marker strongly correlates with NK cell-mediated lysis of target cells [18]. In our experiments, in which we used K562 as target cells, CD107a was expressed at higher levels on the membrane of the cytotoxic NK cell subset (CD16+CD56^dim^) from SAT *versus* PBMC (blood) (even in the absence of stimulation), indicating that NK cytotoxicity was higher in the SAT as compared to the blood (Fig 7A). This result is in contrast with what has been observed in the human visceral AT (omental), in which only CD16-CD56^dim^ NK cells were increased as compared with the peripheral blood [43], but this is not the NK subset showing highest levels of cytotoxicity [20, 44]. The subset of CD16+CD56^dim^ NK cells is also able to release IFN-γ, and in higher amounts as compared to blood-derived NK cells (Fig 7B), thus contributing to local inflammation and IR [45, 46]. We have also measured cytotoxity of CD16+CD56^dim^ NK cells against MΦ from the SAT, using a protocol previously described [47]. Results in Fig 7C show that NK cells in the SAT kill endogenous MΦ (measured by expression of the degranulation marker CD107a), and maybe also other cell types, further inducing local release of “self” antigens.

DNA damage may also represent a mechanism for cell death and “self” antigen release in the SAT (see above, Fig 5C).

### Antibody production occurs in the SAT

B cells promote IR through regulation of T cells [16]. In particular, B cells have been shown to support pro-inflammatory T cells in the context of obesity [10] and T2D [48], and removal of B cells from PBMC significantly reduces T cell secretion of IFN-γ and IL-17 [10]. Moreover, B cells promote IR also through the secretion of pro-inflammatory cytokines and pathogenic autoantibodies [16]. A publication of 2011 has shown that IgG autoantibodies are present in the serum of IR individuals at a significantly higher extent as compared to IS individuals [16]. This result was obtained using a protein array in which more than 9000 selected “self” antigens were probed with serum from 32 IR and 32 IS individuals, overweight to obese, age- and BMI- matched. Results showed 122 IgG autoantibodies segregating with IR and 114 segregating with IS. In both groups the majority of “self” antigens are intracellular proteins (Golgi and endoplasmic reticulum proteins, RNA polymerase, glutathione transferase, signaling proteins) with variable tissue expression. The presence of 114 specificities segregating with IS leaves the mechanism completely open at this point but suggests that some autoantibodies may also be protective. This publication remains the only one to our knowledge that demonstrates that in obese individuals IR is associated with serum autoantibodies directed against specific “self” antigens.

**Fig 8:**
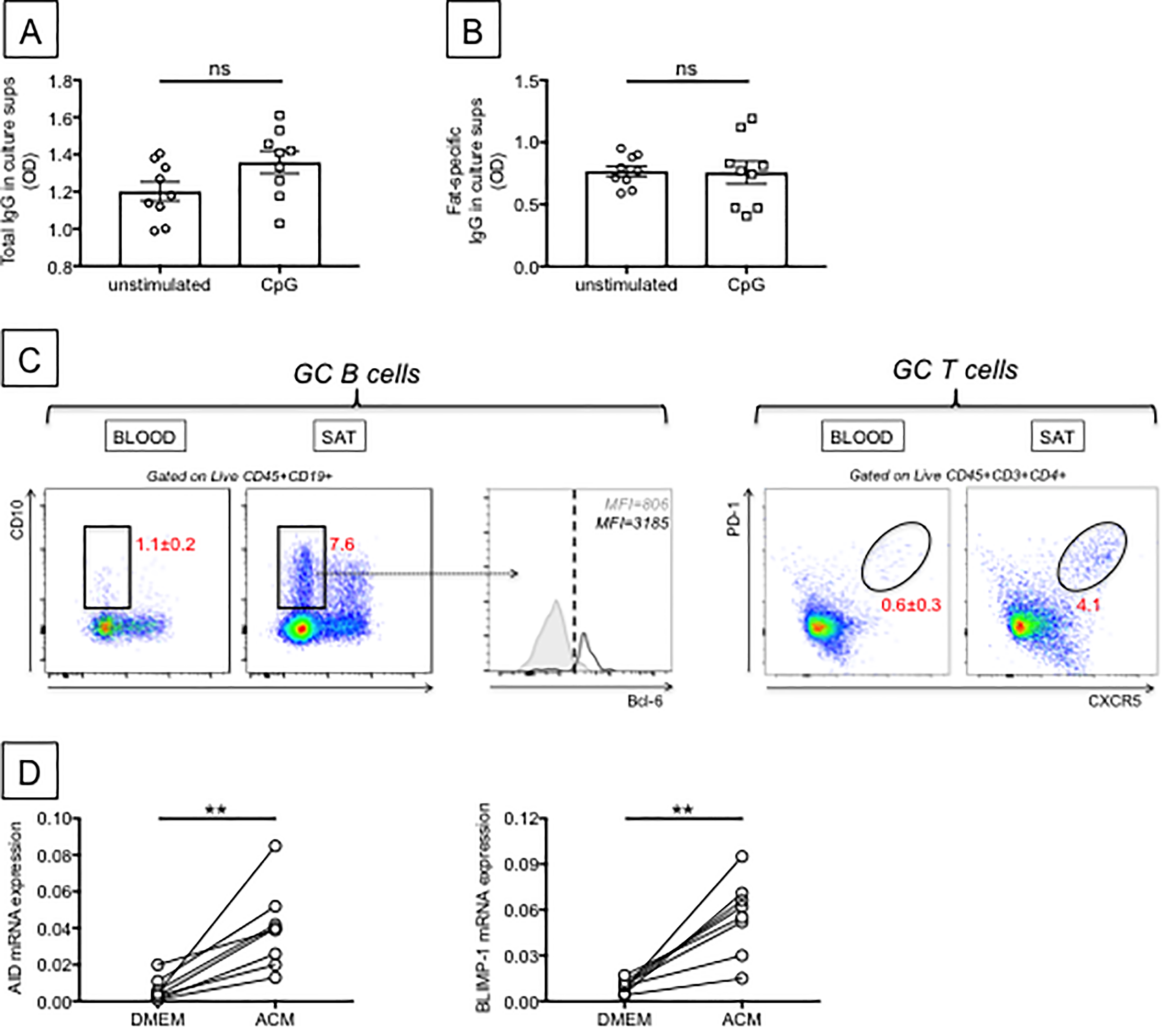
Autoantibody production in the SAT. **A.** Total IgG antibodies were detected by ELISA in supernatants of unstimulated and CpG-stimulated SVF cultures. **B.** Fat-specific IgG antibodies were detected by ELISA after enrichment in IgG antibodies. ELISA plates were coated with protein lysates from adipocytes from the same individuals. **C.** Detection of GC B and T cells in the SVF, and in the blood (from the same individuals) as control, was performed by flow cytometry. GC B cells were CD19+CD10+IgD- and also expressed intracellular Bcl-6. Grey line, filled histogram: blood; black line, unfilled histogram: SVF. GC T cells were CD3+CD4+PD-1+CXCR5+. **D.** Naïve B cells were sorted from the peripheral blood of 8 lean healthy individuals and stimulated in the presence of ACM for 5–8 days to detect mRNA expression of AID and BLIMP-1, respectively. Cells (10^6^/ml of ACM) were stimulated with 5 μg/ml of CpG. Results show qPCR values (2^-ΔΔCt^) of RNA expression of AID (top) and BLIMP-1 (bottom). Mean comparisons between groups were performed by Student’s t test (two-tailed). **p<0.01.

We hypothesize that toxic intermediates generated during aberrant metabolism could induce pro-inflammatory responses and activate B cells to release pathogenic autoimmune antibodies. This supports the importance of understanding antibody specificities. Autoantibodies can be directed against a variety of molecules, such as nucleic acids, lipids, and proteins, and these antigens can be located in the nucleus or cytoplasm, on the cell surface, or in the extracellular milieu [49]. We measured antibody secretion in the supernatants of unstimulated or CpG-stimulated cultures of SVF. Results in Fig 8A show comparable amounts of total IgG antibodies in the supernatants of unstimulated and CpG-stimulated SVF cultures, whereas Fig 8B shows comparable amounts of fat-specific IgG in unstimulated and CpG-stimulated SVF cultures. The major point here is that the lymphocytes are already stimulated in the SVF and the CpG stimulus does not give further stimulation for IgG secretion. These results suggest that the ongoing process of cell death in the SAT due to the mechanisms described above may be responsible for the release of “self” antigens from any cell in the SAT, and these “self” antigens induce chronic stimulation of B cells without the need of additional stimulation. At this point, we do not know the specificity of these autoantibodies. We are planning to characterize antibody specificitites using Mass Spectrometry, in order to identify “self” proteins which may stimulate the secretion of pathogenic antibodies and will be used in protein array experiments. Our data herein, showing autoantibody production in the SAT, demontrates the crucial role of the SAT in the release of these specificities.

B cell activation and production of high affinity antibodies typically require the generation of GCs. During GC reactions, B cells proliferate, undergo clonal expansion, CSR, SHM of VH genes and affinity maturation [50]. Because we have detected (auto)antibody production in the SAT, we performed staining of the SVF to evaluate the presence of GCs in the SVF. Results (Fig 8C) show the identification of GC B cells (CD19+CD10+IgD-), as well as GC T cells (CD3+CD4+PD1+CXCR5+) in the SAT. GC B cells in SVF were also found positive for intracellular Bcl-6. Most of the GC B cells were Dark Zone GC B cells (CD10+CXCR4+) (data not shown), which is the centroblast-rich area proximal to the T cell area [51]. Dark Zone GC B cells up-regulated the expression of genes involved in SHM in response to signals delivered by GC T cells and dendritic cells [52]. Effector cells developing from this interaction are the early extrafollicular plasmablasts, which appear before B cells re-enter the GC, and usually secrete low affinity antibodies, as reviewed in [53, 54]. However, also during this early T-B interaction there is strong induction of CSR with expression of activation-induced cytidine deaminase (AID), the enzyme of CSR and SHM [55].

This study, in which we had the unique opportunity to evaluate GC B cells and GC T cells in the SAT as compared to the blood, is the first to our knowledge to detect GCs in the human SAT. This finding is relevant because competent GC T cells, which have already been shown to accumulate in resting human lymph nodes, provide superior help to B cells for Ig secretion, as compared to those in the peripheral blood [56].

Because we detected GC B cells in the SAT, and we also detected the presence of cytokines promoting isotype class switch in the SAT (see below), we evaluated if the ACM was able to induce mRNA expression of AID, the enzyme of CSR and SHM, and BLIMP-1, the transcription factor involved in plasma cell differentiation [57]. Briefly, we sorted naïve B cells from the peripheral blood of 8 lean healthy individuals and we stimulated them in the presence of ACM and CpG for 5 and 8 days to induce optimal expression of AID and BLIMP-1, respectively. Results in Fig 8D clearly show that the ACM (but not DMEM, negative control) was able to induce mRNA expression of AID and BLIMP-1 and therefore class switch in the SAT. Future experiments will evaluate if AID is localized in Dark Zone GC B cells in the SAT, as well as its intracellular localization (nuclear or cytoplasmic), providing further evidence for CSR in the human SAT.

### Transcription factors for autoimmune antibody production in the SAT

**Fig 9:**
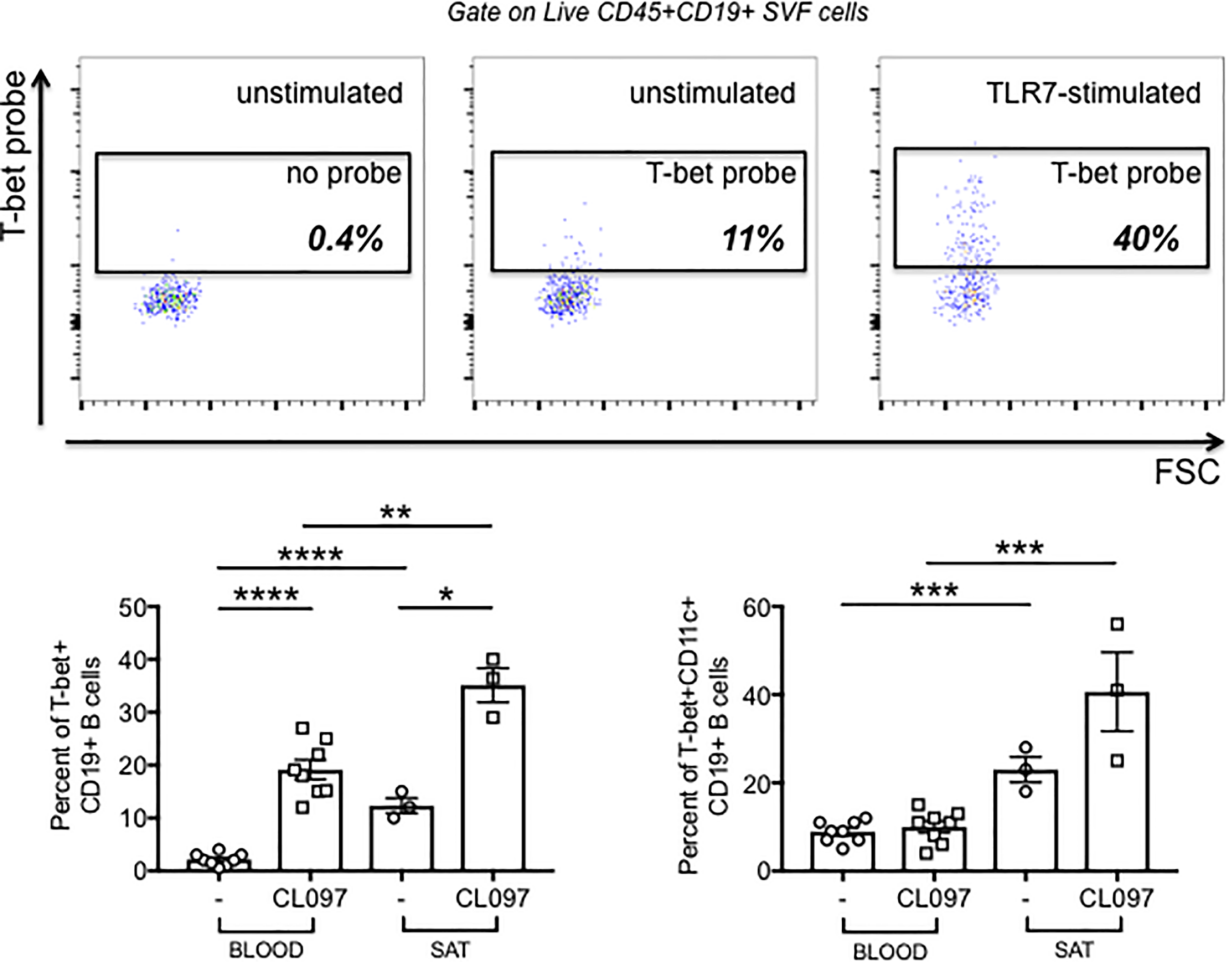
Expression of T-bet and CD11c in B cells from SAT SVF. **A.** Detection of T-bet by Prime Flow. Unstimulated and CL097-stimulated SVF were labeled with Live/Dead detection kit and then with anti-CD19/CD27/IgD antibodies to detect B cells. Negative control was the sample without the labeled probe. Approximately 200,000 cell events were acquired from each sample on the flow cytometer. **Top.** A representative dot plot is shown. **Bottom left.** Means±SE from independent experiments, in which unstimulated and stimulated samples from the SVF and from PBMC (blood) of different obese individuals age-, gender- and BMI-matched were analyzed, are plotted. Mean comparisons between groups were performed by Student’s t test (two-tailed). *p<0.05, **p<0.01, ****p<0.0001. **Bottom right.** Detection of T-bet+CD11c+ B cells. Unstimulated and CL097-stimulated PBMC and SVF were processed as in A, then membrane labeled with anti-CD11c. Mean comparisons between groups were performed by Student’s t test (two-tailed). ***p<0.001.

We measured the expression of the transcription factor T-bet, encoded by the *tbx21* gene, which is known to be associated with the secretion of IgG2a/c antibodies in mice (IgG1 in humans) [58–60], which are pathogenic (autoimmune) antibodies. We measured T-bet expression before and after stimulation with the TLR7 agonist CL097 (single stranded RNA), a good inducer of T-bet. These experiments were done using the PrimeFlow RNA assay. This technique measures intracellular mRNA by an amplified, Fluorescence In Situ Hybridization technique, in combination with flow cytometry and therefore allows the evaluation of T-bet expression in B cells. Results in Fig 9 (top) show that T-bet expression is detectable in both unstimulated and stimulated B cells from the SVF, suggesting that even in the absence of exogenous stimulation (but in the presence of chronic exposure to “self” antigens) T-bet may drive secretion of autoimmune antibodies in the SAT, and we suggest they may be autoimmune antibodies. In blood-derived B cells from obese individuals age-, gender- and BMI-matched, conversely, T-bet expression is barely detectable in unstimulated cells and is increased after TLR7 stimulation Fig 9 (bottom left). T-bet expressing B cells have been found in autoimmune diseases [61]. Fig 9 (bottom right) also shows that T-bet expressing B cells also express CD11c, (Itgax) [58, 60], an integrin involved in autoantigen presentation [61], suggesting that CD11c+ B cells in the SAT may be autoantigen-presenting cells, leading to the secretion of autoimmune antibodies. T-bet+CD11c+ B cells are expanded in healthy elderly individuals [62] and also in patients with autoimmunity, promoting the secretion of anti-chromatin IgG in SLE patients [63].

**Fig 10:**
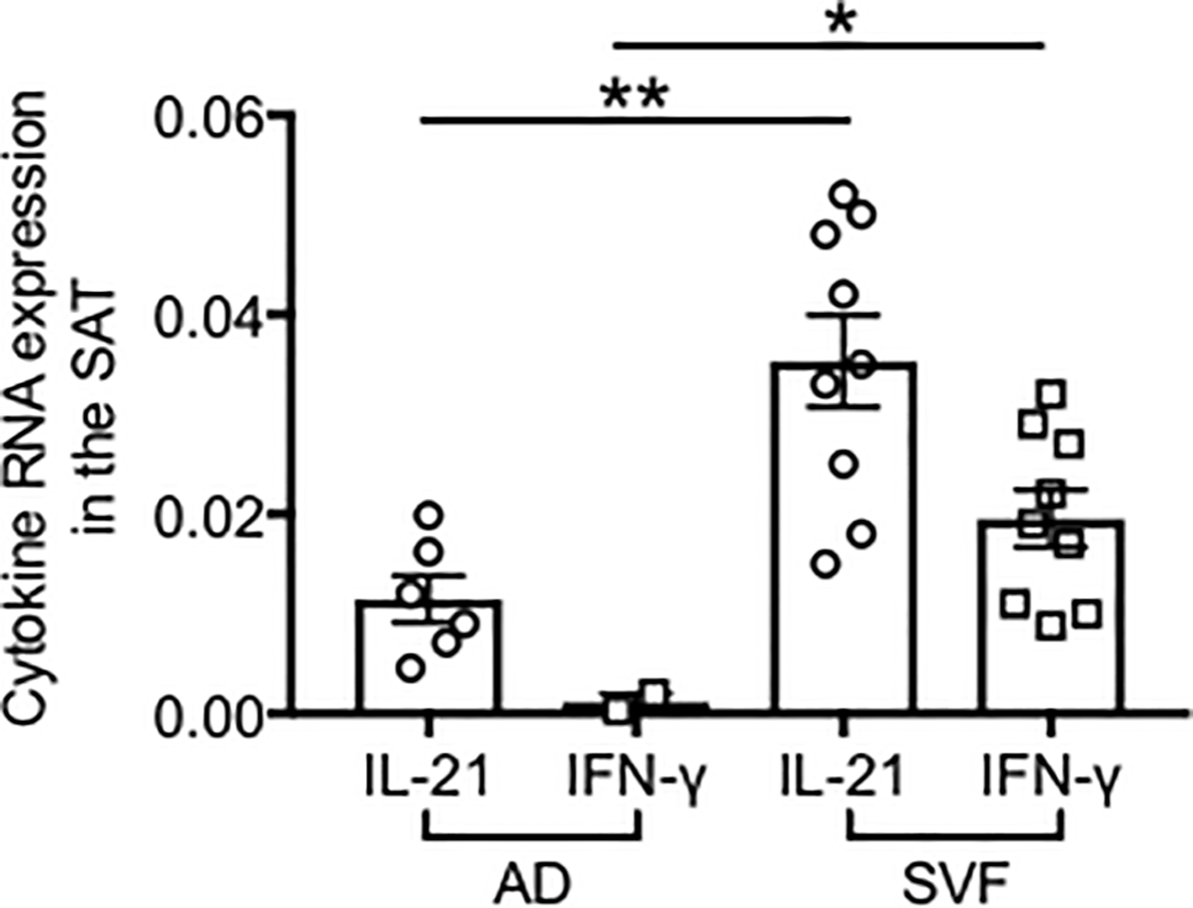
RNA expression of IFN-γ and IL-21 in the obese SAT. Adipocytes (AD) were sonicated for cell disruption in the presence of TRIzol. SVF was also resuspended in TRIzol. AD and SVF were from the same individuals. Results show qPCR values (2^-ΔΔCt^) of IFN-γ and IL-21. Mean comparisons between groups were performed by Student’s t test (two-tailed). **p<0.01, **p<0.01.

It has previously been shown that in both humans and mice T-bet expression is induced by nucleic acids and is regulated by the GC T cell-derived cytokines IL-21, IFN-γ and IL-4 [64], with IL-21 and IFN-γ promoting and IL-4 antagonizing T-bet expression in the context of TLR stimulation [58]. T-bet also promotes IFN-γ production, favoring TH1 differentiation and inflammation [65]. In addition, IL-21 induces Bcl-6 expression on B cells expressing IL-21R [66], Ig production and plasma cell differentiation and its absence leads to impaired GC formation and antibody production [67, 68]. We measured by qPCR the production of these two cytokines in adipocytes and SVF. Results in Fig 10 show that adipocytes express mRNA for IL-21 and IFN-γ, but less than the SVF. No expression of IL-4 was detected (not shown). Moreover, these cytokines were not detected in the blood of either obese or lean individuals (not shown). These results altogether suggest that the SAT provides support to B cells for the secretion of autoimmune antibodies through the secretion of IL-21 and IFN-γ.

## Conclusions

In this study we have shown that immune cells infiltrate the human obese SAT, contribute to local and systemic inflammation as well as to the production of autoimmune antibodies. We have identified several molecules and mechanisms which may be responsible for the release of “self” antigens in the human obese SAT, induce class switch and the production of autoimmune IgG antibodies. These autoantibodies may be pathogenic, as it has already been shown in mice, as they can form immune complexes with “self” antigens, which in turn activate complement and Fc receptors on immune cells, leading to enhanced local inflammation, remodeling of the AT, impairment of adipocyte function and of nutrient metabolism, and exacerbation of obesity-associated conditions. These antibodies can also exert additional detrimental effects systemically targeting distinct clusters of self proteins. To our knowledge, this study is first of its kind to characterize the phenotype and function of immune cells in the human obese SAT, as compared to the blood of the same individuals. Key challenges for the field will be to identify therapeutic strategies of intervention to lose weight, reduce body fat, systemic inflammation and the pathogenic role of immune cells. Importantly, immune responses to fight infections will also be improved.
